# Optical Characteristics and Applications of AIE Racemic C6-Unsubstituted Tetrahydropyrimidines

**DOI:** 10.3389/fchem.2021.800177

**Published:** 2021-11-26

**Authors:** Qiuhua Zhu

**Affiliations:** Guangdong Provincial Key Laboratory of New Drug Screening, School of Pharmaceutical Sciences, Southern Medical University, Guangzhou, China

**Keywords:** tetrahydropyrimidines, aggregation-induced emission, critical micelle concentration, fluorescence probe, fluorescence thermometer, mechanofluoromechanism, endoplasmic reticulum imaging

## Abstract

Racemic C6-unsubstituted tetrahydropyrimidines (THPs) are the products of an efficient five-component reaction that we developed. THPs show strong AIE characteristics, that is, completely no fluorescence in different solvents but strong emission with fluorescence quantum yields (*Φ*_F_) up to 100% upon aggregation. However, the *Φ*_F_ values of their pure enantiomers are lower than 46%. Unlike common AIE compounds with crowded aryl rotors on a π-bond or on an aryl ring, THPs have three completely non-crowded aryl rotors on a non-aromatic chiral central ring (tetrahydropyrimidine). In this mini review, we first discuss the AIE characteristics of THPs and the influences of molecular structures on their molecular packing modes and optical properties, and then present their applications and forecast the development of other racemic AIE compounds.

## Introduction

**FIGURE 1:**
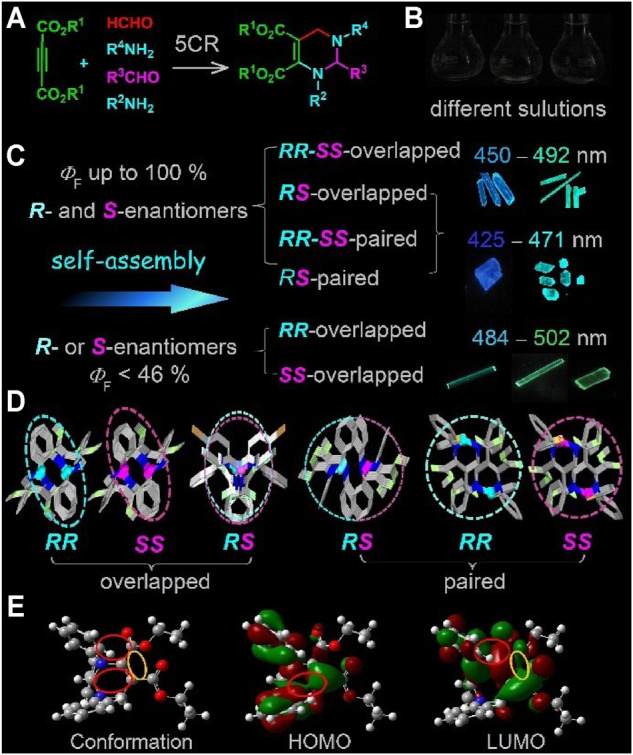
Synthesis of tetrahydropyrimidines (THPs) and their aggregation-induced emission (AIE) characteristics. **(A)** The 5CR for the synthesis of THPs (Zhu et al., 2013). **(B)** Photos of THPs in *n*-hexane (Zhu et al., 2019), cyclohexane (Zhu et al., 2015), and ethanol (Zhu et al., 2013). **(C)** *R*- and *S*-enantiomer packing modes of THPs and their influence on fluorescence quantum yields and emission wavelengths. **(D)** Examples of *R*- and *S*-enantiomer packing modes in THP single crystals (Zhu et al., 2019). Reproduced with the permission of Elsevier. **(E)** Through-space conjugation (marked in color circles) in the HOMO and LUMO calculated from the conformation of THP-1c crystal (Zhu et al., 2015). Reproduced with permission from the Royal Society of Chemistry. The photos in **(B)** and **(C)** were taken under a 356 nm light.

Organic fluorescent materials have attracted great interest owing to their wide applications. However, conventional organic fluorophores are limited in their uses because they emit weak or even no fluorescence upon aggregation in spite of high emission in dilute solutions. This thorny problem has been fundamentally solved since the unusual aggregation-induced emission (AIE) phenomenon, that is, non-emission in solution but strong fluorescence upon aggregation, was found and termed by the Tang group in 2001 (Luo et al., 2001). In recent years, AIE fluorophores have attracted lots of attention from scholars in different research fields (Liang et al., 2015; Mei et al., 2015; Li et al., 2020; Song et al., 2020; Wang et al., 2020; Xu et al., 2020; Li et al., 2021), such as theranostic applications (Song et al., 2020), AIE-active gels (Li et al., 2021), chemo/biosensors (Liang et al., 2015), and bio-imaging (Qi et al., 2018; Lin et al., 2019; Xu et al., 2021). AIE molecules have a common structural feature: nonplanar and flexible. Most of the AIE fluorophores have crowded aryl rotors on a π-bond or on an aryl ring, for example, propeller-like 1-methyl-1,2,3,4,5-pentaphenylsilole (Luo et al., 2001) and tetraphenylethylene (TPE). In 2011, we developed an efficient five-component reaction (5CR) (Figure 1A) for the synthesis of a series of new racemic C6-unsubstitutied tetrahydropyrimidines (THPs) with strong AIE characteristics (Figures 1B, C) (Zhu et al., 2013). Racemic THPs show non-fluorescence in different solvents but strong emission with fluorescence quantum yields (*Φ*_F_) up to 100% upon aggregation (Zhu et al., 2015; Zhu et al., 2019). However, the *Φ*_F_ values of their pure enantiomers are lower than 46% (Zhu et al., 2017). Unlike AIE compounds, THPs have no aryl rotor on a π-bond or on an aryl ring. They have a non-aromatic chiral central ring (tetrahydropyrimidine), on which there are three completely non-crowded and freely rotatable aromatic rings. In this mini review, we first discuss the AIE characteristics of racemic THPs and the influences of molecular structures and hetero-enantiomer molecular packing modes on their optical properties, then, their AIE mechanism, other optical properties, and applications. In the last, the future development and application of other racemic AIE compounds have been forecasted.

## Aggregation-Induced Emission and Influence Factors on Optical Properties 

THPs were synthesized by the simple and efficient 5CR using easily available reactants of but-2-ynedioate, aniline, formaldehyde, and aromatic aldehyde as reactants (Zhu et al., 2013) (Figure 1A). As shown in Figures 1B, C, racemic THPs show strong AIE properties (Zhu et al., 2013; Zhu et al., 2019). They are completely nonemissive in different solvents, from nonpolar solvents, polar solvents, to proton solvents. However, they show strong fluorescence upon aggregation. The *R-* and *S*-enantiomers of racemic THPs have been found to self-assemble in four modes: *RR/SS*-overlapped, *RS*-overlapped, *RR/SS*-paired, and *RS*-paired packing modes (Zhu et al., 2019) (Figure 1D). The overlapped mode means that one or two of the three aryl substituents are overlapped, the paired mode represents that there are no overlapped aryl substituents.

The fluorescence peak wavelengths (*λ*_em_) of racemic THPs mainly depend on their *R-* and *S*-enantiomer packing modes and are influenced to a certain extent by substituents R^2^ and R^4^ (Zhu et al., 2015; Zhu et al., 2019). The dihedral angle (*α*) between the R^2^ phenyl and –C=C– plane is 31°–37° in the in *RS*- and *RR*/*SS*-overlapped modes but larger (43°–59°) in *RS*- and *RR*/*SS*-paired modes. For the THPs with the same molecular structure, the *λ*_em_ values of their polymorphs are in an order: *RR/SS*-overlapped mode > *RR/SS*-paired mode **>** *RS*-paired mode or *RS*-overlapped mode. Under the same molecular packing mode, aromatic R^2^ and R^4^ with electron-donating/withdrawing substituents caused a red/blue shift in *λ*_em_, but R^1^ and R^3^ showed almost no influences on *λ*_em_. Substituent R^3^ displays a great influence on the *R-* and *S*-enantiomer packing modes of racemic THPs. For example, THPs with R^3^ = Ph or atom-substituted Ph such as 4-BrPh can form *RR*/*SS*-overlapped mode, and some of them can also form *RS*-overlapped, *RS*-paired, or *RR*/*SS*-paired polymorphs. However, THPs with R^3^ = group substituted phenyl (4-CN/4-MeO/3-MeO-4-OHPh) are arranged only in *RS*- overlapped or *RS*-paired mode with shorter *λ*_em_ values.

The fluorescence quantum yields (*Φ*_F_) of THPs mainly depend on the combinations of substituents R^1^ − R^4^ rather than the enantiomer packing modes. The influence of substituents R^1^ − R^4^ on the *Φ*_F_ values of THPs could not be explained by electronic effects (Zhu et al., 2015; Zhu et al., 2019). In our previous work (Zhu et al., 2019), the optical properties of 66 racemic THP different substituents R^1 ^− R^4^ in *n*-hexane solutions are completely nonemissive and have similar absorption wavelengths (*λ*_ab_) (315–320 nm) except those with R^2^ = R^4^ = alky (*λ*_ab_ = 293 nm). However, upon aggregation, only two THPs with aliphatic R^2^ and R^4^ show no fluorescence at all, other 64 THPs with aromatic R^2^ and R^4^ are emissive and display different fluorescence properties with excitation wavelengths (*λ*_ex_) equal to 340–409 nm, emission wavelengths (*λ*_em_) equal to 425–494 nm, and fluorescence quantum yields (*Φ*_F_) equal to 3%–100% (nine THPs with *Φ*_F_ = 90%–100%, 11 THPs having *Φ*_F_ = 80%–89%, and 33 THPs with *Φ*_F_ = 50%–79%). The solid-state *Φ*_F_ values of THPs could be significantly changed by simple changes in the combinations of R^1^ − R^4^. The 20 THPs with *Φ*_F_ > 80% were mainly from the combinations of R^1^ = Me, R^2^ = R^4^ = Ph, and R^3^ = aromatic heterocycles (such as electron-deficient pyridin-4-yl and electron-rich thiophen-2-yl) or various substituted phenyls (such as 4-BrPh, 4-CNPh, 4-ClPh, and 4-MeOPh); the combinations of R^1^ = Me, R^2^ = R^4^ = 3-CF_3_/4-ClPh/3-BrPh, R^3^ = Ph/3-CF_3_Ph/4-CF_3_Ph; and the combinations of R^1^ = Et, R^3^ = Ph, and R^2^ = R^4^ = Ph/4-ClPh or R^2^/R^4^ = Ph/4-Br or 3-CF_3_Ph/Ph. It is worth mentioning that the design of THPs with R^2^ = R^4^ was preferred because the 5CR for synthesis of THPs might produce two position isomers when R^2^ ≠ R^4^, and the isomers are usually difficult to be isolated.

The influences of hetero-enantiomers and pure-enantiomer packing modes on the optical properties of THPs were investigated via the optical properties of four racemic THPs and their pure enantiomers in crystals (Zhu et al., 2017). The pure enantiomeric THPs also show AIE characteristics, and the *R*- and *S*-enantiomers in pure enantiomeric THPs are aligned in *RR*- or *SS*-overlapped mode, which is the same as that in their racemic aligned in *RR/SS*-overlapped mode. However, unexpectedly, the *Φ*_F_ values (25%–46%) of the seven pure enantiomers in aggregates are much lower than those (48%, 80%, 93%, and 100%, respectively) of their corresponding enantiomers. The difference between the *Φ*_F_ values of enantiomeric and racemic THPs mainly arise from their difference in nonradiative rate constants (*k*_nr_): The *k*_nr_ values of enantiomeric THPs are much larger than those of their corresponding racemic THPs. Since the conformations and *R-* or *S*-alignments in pure enantiomeric THPs are almost the same as those in racemic THPs, the larger *k*_nr_ values of enantiomeric THPs are expected to relate to their different general molecular alignments and chiral space group. The *λ*_em_ values (484–502 nm) of the seven pure enantiomers packed in *RR*- or *SS*-overlapped mode are the same as or slightly longer than those (472–488 nm) of the corresponding racemics packed in *RR*-*/SS*-overlapped mode.

## Aggregation-Induced Emission Mechanism

Since the advantages of AIE fluorophores in wide application areas and the complex interactions in aggregates, the AIE mechanisms were deeply studied, and different mechanisms have been proposed (Chen et al., 2019; Peng and Shuai 2021; Rodrigues and Melo 2021), for example, restriction of intramolecular rotation (RIR) (Chen et al., 2003; Zeng et al., 2007), restriction of intermolecular motion (RIM) (Mei et al., 2015), minimum energy conical intersection (MECI) (Sasaki et al., 2016), suppression of Kasha’s rule (Qian et al., 2017), excited-state double-bond torsion (Zhang et al., 2019), etc. Although some debate still exists, restriction of intramolecular motions (RIM) has been generally accepted as central to the AIE working mechanism (Chen et al., 2019).

Since molecular structure is a main factor influencing optical properties, the influences of intermolecular interactions, molecular alignments, and conformations on the fluorescence properties of THPs should be studied under no change in molecular structure. Considering the polymorphs have the same molecular structure, and some of THPs could easily form polymorphs, we explored the AIE mechanism of racemic THPs via the fluorescence properties of seven crystalline polymorphs of three THPs (Zhu et al., 2015). Fluorescence quantum yields depend on the radiative rate constant (*k*_r_) and the nonradiative rate constant (*k*_nr_). Much larger *k*_r_ than *k*_nr_ would lead to high fluorescence efficiency; on the contrary, much larger *k*_nr_ than *k*_r_ would lead to practically no emission. Although THPs have low conjugated molecular structure, the theoretical calculation of HOMOs and LUMOs based on the molecular conformations in these polymorphs indicate that THPs possess unusual through-space conjugation (marked in color circles in Figure 1E), radiative-favored intercrossed local excitation (LE) and intramolecular charge transfer (ICT) excitation states (Li et al., 2012), hence, their radiative rate constants (*k*_r_) (0.41–1.03 × 108 s^−1^) are as large as those of conventional π-conjugated fluorophores (Choi et al., 2014; Yagai et al., 2014). However, the *k*_nr_ values in the polymorphs with the same molecules are significantly different (0.05–3.48 × 108 s^−1^). This is because the molecular structures of THPs are highly flexible (the freely rotatable single C–C and C–N bonds, as well as the non-rigid aliphatic central ring), and the restriction extents of molecular motion, that is, the *k*_nr_ values, are sensitive to the changes in their intra- and intermolecular interactions. The short-range interaction between the intramolecular aryl R^2^ and R^4^ proves to be an important factor influencing *k*_nr_ values: the shorter the distance between intramolecular aryl R^2^ and R^4^, the smaller the *k*_nr_ value. In addition, racemic THPs generally have similar *k*_r_ values to those of their corresponding pure enantiomers, but their *k*_nr_ values are much smaller than those of their corresponding pure enantiomers. This means that heteroenantiomeric self-assembly can efficiently suppress the nonradiative decay process of THPs. Therefore, the strong AIE of THPs arises not only from the restriction of intramolecular motion as common AIE compounds but also from unusual through-space conjugation, radiative-favored inter-crossed LE and ICT excitation states, as well as heteroenantiomeric self-assembly.

## Applications of Racemic Tetrahydropyrimidines

**FIGURE 2:**
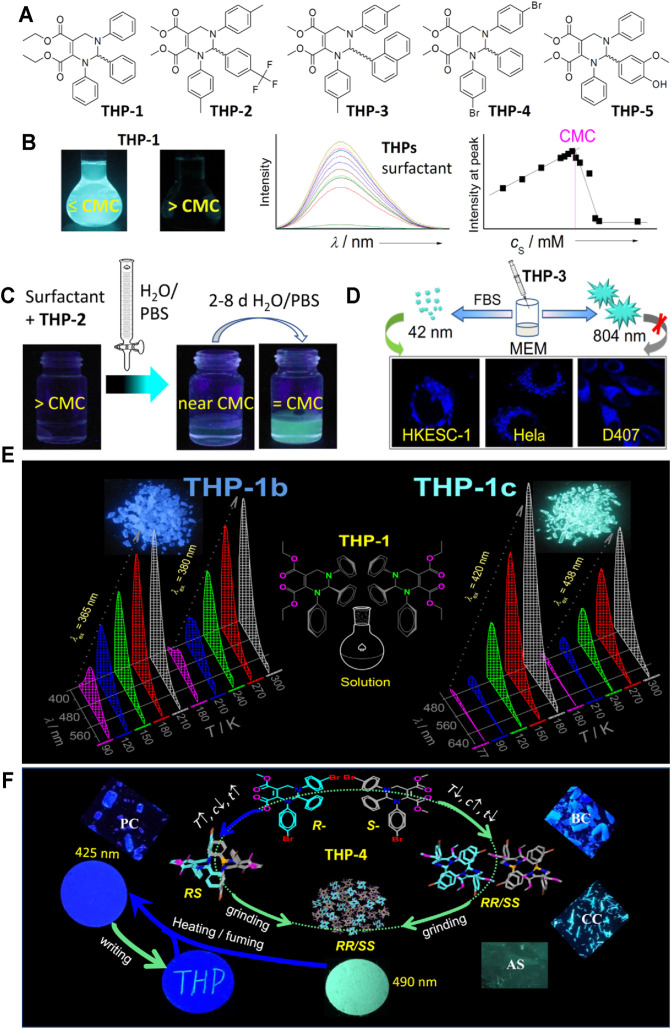
Applications of some THPs based on AIE and on AIE and other characteristics. **(A)** Molecular structures. **(B)** Photos of **THP-1** in surfactant solutions with concentration lower than or equal to critical micelle concentration (CMC) and higher than CMC, respectively. The emission spectra of THPs in different concentrations of surfactant solutions as well as the relationship between the fluorescence intensity of THPs at peak (Zhu et al., 2014; Cai et al., 2015). Reproduced with permission from the Royal Society of Chemistry. **(C)** Schematic process of **THP-2** as fluorescence indicator for CMC titration (Wu et al., 2020). Reproduced with the permission of American Chemical Society. **(D)** Schematic preparation of **THP-3** nanoparticles by adding 10% fetal bovine serum (PBS) in cell culture medium minimum essential medium (MEM) and the confocal microscopic imaging of living HKESC-1, Hela, and D407 cells incubated with the as-prepared **THP-3** nanosuspension (Zheng et al., 2018). Reproduced with the permission of Elsevier. **(E)** Photos of **THP-1** in polymorphs **1b** and **1c**, and their emission spectra at different temperatures in different temperature ranges excited at different wavelengths (365 and 380 nm for **1b**, and 420 and 438 nm for **1c**) (Zhu et al., 2016). Symbols b and c represent blue and cyan fluorescence, respectively. Reproduced with permission from the Royal Society of Chemistry. **(F)** Schematic formation process of **THP-4** polymorphs by different packing modes of *R*- and *S*-enantiomers, and the mechanofluoromatic characteristics and use of **THP-4** as erasable material (Liu et al., 2017). PC, purple crystals; BC, blue crystals; CC, cyan crystals; AS, amorphous state. All photos were taken under a UV light (365 nm). Reproduced with the permission of Elsevier.

In addition to AIE characteristics, THPs were found to have other unusual characteristics. Figure 2 displays the applications of THPs 1–5 based on their AIE or on AIE and other characteristics.

### CMC Determination Based on the Characteristics of Strong AIE in Surfactant Dilute Solutions but Non-Emission in Surfactant Micelles

Surfactants have wide applications because of their diverse properties, such as dispersion, solubilization, wetting, emulsification, foaming, and corrosion resistance. They begin to form thermodynamically stable micelles when their concentrations are higher than a particular concentration called critical micelle concentration (CMC) and show sharp changes in properties. Surfactant CMC values are usually influenced by environmental factors and relates to suppliers (Cai et al., 2011). Therefore, simple and fast CMC determination is usually needed, which leads to the development of different CMC determination methods (Kalyanasundaram and Thomas 1977; Karukstis and Gulledge 1998; Nichols and Kindt 2002; Cai et al., 2011; Hussain et al., 2018; Scholz et al., 2018), such as electric conductivity (Scholz et al., 2018), absorbance spectra (Hussain et al., 2018), and fluorescence (Hussain et al., 2018; Scholz et al., 2018) methods. Compared with other methods, the method based on the change in fluorescence intensity has clearly visible and highly sensitive advantages. Therefore, we explored the application of THPs as CMC probes. We found that THPs (for example, THPs **1**–**5** in Figure 2A) had no emission in ionic surfactant micelles but emit strong fluorescence in dilute ionic surfactant solutions (photos in Figure 2B). However, conventional fluorophores in micelles generally show stronger fluorescence intensity than fluorescence in surfactant solutions (Wandruszkav 1992). Based on the unusual characteristics of THPs in surfactant solutions, we developed **THP-1** as a new type of fluorescence-turn-on probe for CMC determination of ionic surfactants (Zhu et al., 2014) and realized high-throughput CMC determination using highly sensitive THPs (Cai et al., 2015). The change in the fluorescence intensity of THPs near CMC is so sensitive that the change can be clearly observed by the naked eye.

Although THPs are excellent probes for CMC, the precise CMC determination is cumbersome, time- and sample-consuming. As shown in Figure 2B, at least 10 samples containing different surfactant concentrations and a certain amount of THP are needed to be prepared and measured, and then the precise CMC value was obtained by the relationship between the surfactant concentration and fluorescence intensity at peak. If THPs could be used as indicators for simple and fast CMC titration, these thorny problems will be solved. In 1946 and 1947, the CMC values of cationic/anionic surfactants were determined by simple and fast titration method using anionic/cationic dyes as indicators (Corrin et al., 1946; Corrin and Harkins 1947; Corrin and Harkins 1947). However, the CMC titration method has not been applied by others owing to the shortcomings of these dyes: very narrow application range (one indicator only suitable for cationic or anionic surfactants), difficulty in observing endpoint change in color or fluorescence intensity (the signal change must be compared with blank through the flask neck), especially needing different concentrations of the indicator (10–300 µM) for different surfactants.

As an ideal indicator for CMC titration, its signal change at CMC demands to be not only clearly visible but also sensitive and prompt. We screened these THPs reported in our previous work (Cai et al., 2015). Pleasantly and unexpectedly, **THP-2** (Figure 2A) proved to be an excellent CMC titration indicator not only suitable for ionic surfactants but also suitable for nonionic surfactants (Figure 2C) (Wu et al., 2020). This is because **THP-2** possesses the unique and excellent characteristics in different surfactant solutions: Above CMC, it dissolves in micelles and shows no emission; it was not until near/at CMC that it is released from micelles and instantly forms aggregates with strong fluorescence. The CMC titration can be conducted in surfactant water (H_2_O) or phosphate buffer saline (PBS) solutions. It is worth mentioning that since the fluorescence change of **THP-2** at CMC is highly sensitive and clearly visible, it is not necessary to keep the concentration of **THP-2** unchanged. Thus, pure water or PBS solution could be used as diluting solution for CMC titration. The concentration of **THP-2** at the titration endpoint is only about 1 µM in zwitterionic surfactant titrated solutions and 2.5 µM in other kinds of surfactant titrated solutions. It is very important that the CMC values, determined by simple, fast, and economical **THP-2**-based titration method, are consistent with those determined by cumbersome, time- and sample-consuming fluorometric method.

### Endoplasmic Reticulum Imaging in Living Cells Based on Strong Aggregation-Induced Emission

Fluorescent probes for specific organelles have attracted more and more attention because of their advantages in tracking the structural and functional changes of organelles (Zhu et al., 2016; Gao et al., 2019; Kubiasova et al., 2020; Yin et al., 2021), which are very important for exploring biological processes and treating diseases. In eukaryotic cells, the endoplasmic reticulum (ER) is an important dynamic organelle involved in many key cellular processes, including calcium storage, lipid metabolism, protein synthesis, and folding (Borgese et al., 2006; Brodsky and Skach 2011; Westrate et al., 2015; Zhang and Hu 2016). Observing the changes in various metal ions, active substances, and the microenvironment in the ER is crucial for diagnosing and treating many diseases. Recently, fluorophores targeting the ER in living cells provide a powerful tool for this observation (Lu et al., 2019; Zhang et al., 2019; Kubiasova et al., 2020; Peng et al., 2020; Danylchuk et al., 2021; Guo et al., 2021; Qiao et al., 2021; Yue et al., 2021; Zhang et al., 2021). However, reported ER-targeted probes more or less have some disadvantages, such as cytotoxicity, rapid metabolism, low signal-to-noise ratio, and rapid photobleaching. 1n 2018, we found that **THP-3** could be used as an excellent dye targeting ER (Zheng et al., 2018). Since **THP-3** is insoluble in water, its nanosuspension was prepared by cell culture medium, minimum essential medium (MEM) with 10% FBS (fetal bovine serum), and the as-prepared nanosuspension was directly used as culture cell medium for ER imaging (Figure 2D). Compared with existing ER-targeted organic fluorescent probes, **THP-3** shows the advantages of low-cost, long-term staining (the fluorescence of **THP-3** could be still clearly observed in the cells after incubated after 48 h), good photostability (the imaging scope keeps the same, and the fluorescence intensity shows no significant change after continuous irradiation of 15 min), high signal-to-noise ratio, and excellent biocompatibility (**THP-3** has no cytotoxicities for all three kinds of investigated cells incubated with 5–40 µM of **THP-3** for 24 and 48 h), which make it a potential specific probe for real-time ER imaging.

### Unusual and Sensitive Temperature-Range-Tunable Fluorescence-on–off Thermometers Based on Aggregation-Induced Emission and Temperature-Range-Tunable Fluorescence Characteristics

Temperature is an important parameter affecting chemical, physical, and biological changes. Therefore, temperature measurement needs to be carried out in almost all different fields, from daily life, industrial, and agricultural production to scientific research. Fluorescent temperature probes have the advantages of ultra-high sensitivity, fast response, high spatial resolution, safe, and noninvasive detection signal. Different kinds of fluorescent thermometers have been developed (Wang et al., 2013; Li et al., 2014; Wu1 et al., 2017; Qin et al., 2018; Kniec et al., 2019; Gao et al., 2020; Meng et al., 2020; Song et al., 2020; Wang et al., 2020; Wang et al., 2020; Back et al., 2021; Wang et al., 2021). However, conventional fluorescent thermometers are sensitive only in a narrow temperature range, which limits their application to a certain degree. Several methods was reported to prepare fluorescence thermometers sensitive in different temperature ranges, such as altering the size of doped semiconductor nanocrystals (Vlaskin et al., 2010), combining different molecular beacons on gold nanoparticles (Ebrahimi et al., 2014), and altering the solvent ratios containing thermometers (Song et al., 2020), which did not really solve the problem.

In 2016, we found that **THP-1** could show highly sensitive fluorescence-color/-on–off changes in different temperature ranges (70–100, 120–150 … 230–260, 270–300 K) only by changing the excitation wavelength (Zhu et al., 2016). As shown in Figure 2E, **THP-1** could form two polymorphs (**1b** and **1c**) emitting blue and green fluorescence, respectively. **THP-1b** and **1c** have high fluorescence quantum yields (72% and 93%, respectively) (Zhu et al., 2015). **THP-1b** exhibits interesting temperature-range-tunable (TRT) changes in fluorescence intensity and wavelength. For example, when excited at 365 and 380 nm, respectively, **THP-1b** shows significant changes in fluorescence intensity and wavelength (from 495 to 433 nm or from 506 to 434 nm) in the temperature range of 90–210 and 180–300 K, respectively. The ratio of the fluorescence intensity at 434 nm to the fluorescence intensity at 550 nm shows a very good linear relationship with temperature. Polymorph **THP-1c** only shows reversible and sensitive TRT fluorescence-on–off change. For instance, when excited at 420 and 438 nm, respectively, **THP-1c** keeps its *λ*_em_ = 496 nm and only displays sensitive fluorescence-on–off change in temperature ranges of 77–180 and 180–300 K by excitation at 420 and 445 nm, respectively. The fluorescence intensity of **1c** at 496 nm shows good functional relationship with temperature. These TRT thermo-sensitive changes of **1b** and **1c** are reversible. This means that **THP-1b**/**1c** can be used as sensitive TRT ratiometric fluorescence/fluorescence-on–off thermometers. The ratiometric fluorescence change is very important in analysis because the detection of fluorescence intensity (*I*_F_) at two or more emission wavelengths allows avoiding many problems existing in simple intensity sensing (Demchenko 2014). Since the fluorescence intensity of **THP-1b**/**1c** in different temperature ranges is proportional to temperature, which is reverse to common fluorescent thermometers, wherein their fluorescence intensity inversely depend on temperature, **THP-1** can be combined with common fluorescent thermometers to prepare sensitive ratiometric thermometers. The sensitive thermo-stimulated fluorescence-color/on–off switching of **1b**/**1c** proved that it comes from the normal thermal expansion and contraction of aggregates rather than the reported thermo-stimulated change in molecular packing modes (Davis et al., 2004; Mutai et al., 2005; Zhang et al., 2012).

### Erasable Material Based on Aggregation-Induced Emission and Mechanofluorochromic Characteristics

Mechanofluorochromism (MFC) means that the fluorescence color and intensity of the materials change obviously under external stimuli, such as shearing, grinding, or pressing, and revert to the original states upon solvent fuming or heating. These materials have attracted increasing attention owing to their potential uses, such as sensors, memory chips, and security inks (Chi et al., 2012; Hayashi et al., 2018; Zhao et al., 2018; Liu et al., 2019; Sharber et al., 2019; Kuimov et al., 2020; Zhang et al., 2021). Many AIE materials were found to possess MFC (Chi et al., 2012; Zhao et al., 2018; Zhao et al., 2018; Kuimov et al., 2020). Although many AIE compounds possess MFC, a few of them show more than 60 nm change in *λ*_em_. **THP-4** was found to show reversible MFC with the change in *λ*_em_ up to 75 nm (Liu et al., 2017). As depicted in Figure 2F, **THP-4** can form four polymorphs: purple crystals (PC), blue crystals (BC), cyan crystals (CC), and amorphous state (AS) under different conditions. A slower crystallization rate (at higher temperatures or lower concentrations) favored the formation of PC with *λ*_em_ = 425 nm. In contrast, a faster rate (at lower temperatures or higher concentrations) favored the formation of BC, CC, and AS with *λ*_em_ = 445, 468, and 486 nm, respectively. The *R*- and *S*-enantiomers in PC of **THP-4** was proven to arrange in *RS*-paired mode, and in *RRSS*-overlapped mode in other aggregate forms. All the four forms of **THP-4** could be changed into the green powder with *λ*_em_ = 490 nm by grinding. The green ground powder would be switched into purple ground powder with *λ*_em_ = 425 nm by heating or solvent fuming. The purple ground powder loaded on a filter paper can be used as excellent erasable material. For conventional mechanofluorometric fluorophores, the mechano-stimulated change in *λ*_em_ arises from changes in molecular alignments or conformations. However, the mechano-stimulated fluorescence change in **THP-4** was demonstrated to originate from the conversion of paired and unpaired enantiomer alignment modes. Interestingly, the cyan fluorescence (*λ*_em_ = 481 nm) of the powder ground from **THP-1b** can spontaneously recover to the original blue fluorescence (434 nm) (Liu et al., 2021). The mechano-fluorochromic mechanism of **THP-1b** is similar to that of **THP-4**, that is, from the conversion of paired and unpaired *R*- and *S*-enantiomer alignment modes and its self-recoverable mechanofluorochromism relates to intermolecular hydrogen bonds.

### Copper (II) Probe Based on the Characteristics of Aggregation-Induced Emission and Copper-Induced Fluorescence Enhancement Along With Particle-Size Decrease

The parameters *λ*_em_ and *Φ*_F_ are two important parameters for evaluating the optical properties of fluorescent compounds. They are not only related to the molecular stacking mode but also to aggregate sizes (Fu and Yao 2001; Fu et al. 2002; Xiao et al. 2003; Xiao et al. 2004). Therefore, controlling the particle sizes of organic fluorophores is important to the development and application of fluorophores. Copper, an important trace element in life systems, plays a very important role in different cell physiological processes (Millhauser 2004; Lutsenko et al., 2007). In normal organisms, the average concentration of blood copper is 15.7–23.6 µM (Jung et al., 2009). Because too much or too little copper will lead to biological metabolic disorder, it is necessary to track or detect its concentration. In 2013, we found that the fluorescence intensity of **THP-5** increased upon adding Cu^2+^ with a good linear relationship between the fluorescence intensity in the range of 0–80 µM of Cu^2+^ (Huang et al., 2013). The fluorescence response to Cu^2+^ is highly selective over other common transition metal ions. Most importantly, the suspension particle sizes of **THP-5** were found to gradually decrease from 1,106 to 255 nm when the concentration of Cu^2+^ increased from 0 to 300 µM. This means that the sizes of **THP-5** in aggregates could be controlled by adding copper ions. Based on the molecular alignment in **THP-5** single-crystal and the obtained experiments, the copper-induced fluorescence enhancement and particle-size decrease are expected to result from the coordination-induced dissociation of intermolecular O–H … O bonds. In addition, **THP-5** showed almost no cytotoxicity in EC109 cells incubated with **THP-5** for 48 h at concentrations below 30 µM. These useful and interesting properties of **THP-5** are expected to be very helpful for new probe design and practical applications in organic particle size.

## Conclusion

THPs are a series of novel excellent fluorophores with multi optical characteristics, strong AIE (completely nonemissive in different solutions but strong fluorescence upon aggregation), nonemissive in surfactant micelles, sensitive mechanofluorometrism based on conversion of *R*- and *S*-enantiomer packing modes, and unusual TRT characteristics. These interesting characteristics are expected to be potentials in different applications. We have found that THPs can be used as excellent targeting ER dyes, unusual CMC probes, TRT thermometers, etc. Unique properties usually correlate with unique molecular structures or alignments. The molecular structures and alignments of THPs have these characteristics: 1) highly stereo and flexible: a flexible and no aromatic central heterocycle, on which three rotatable aryls are not conjugated and arrange in completely different spatial directions (up, down, and in front of the central ring); 2) good electronic conjugation formed via through-bond and through-space pathways; 3) there is a chiral carbon in the flexible central ring, and the alignments of the *R*- and *S*-enantiomers in racemic THPs could restrict molecular motions much more efficiently than the alignments of pure *R*- or *S*-enantiomers in pure chiral THPs, which leads to much higher fluorescence quantum yields of racemic THPs than pure enantiomeric THPs; 4) The *R*- and *S*-enantiomer packing modes could be easily controlled and lead to polymorphs with significantly different fluorescence colors. The efficient methods of enhancing *Φ*_F_ values and preparing polymorphs with significantly different fluoresce wavelengths by simple heteroenantiomeric self-assembly are first reported and expected to be suitable for other organic compounds rather than only THPs. Because organic racemics can be easily prepared by chemical synthetic methods, this mini review is expected to encourage the development and application of new racemics with multifluorescent characteristics.
